# Causal Relationship Between Circulating Omega‐3 Fatty Acid and Cerebral Small Vessel Disease: A Mendelian Randomization Study

**DOI:** 10.1002/fsn3.71344

**Published:** 2025-12-15

**Authors:** Yaodan Zhang, Lingxue Gong, Xinmin Deng, Jingtao Liang, Xiaofeng Lv, Rui Lai, Meijun Liu

**Affiliations:** ^1^ Department of Neurology Hospital of Chengdu University of Traditional Chinese Medicine Chengdu China; ^2^ Clinical Medical College Guizhou Medical University Guiyang China; ^3^ School of Acupuncture and Moxibustion Chengdu University of Traditional Chinese Medicine Chengdu China

**Keywords:** causal relationship, cerebral microbleed, cerebral small vessel disease, omega‐3 fatty acid

## Abstract

Cerebral small vessel disease (CSVD) plays an important role in the onset of stroke and cognitive dysfunction. Its primary imaging manifestations encompass lacunar stroke (LS), white matter hyperintensity (WMH), white matter perivascular space (WMPVS), and cerebral microbleed (CMB). While omega‐3 fatty acid (FA) has shown potential protective effects against vascular diseases, their causal role in CSVD remains unclear. This study aims to investigate the causal relationship between circulating Omega‐3 FA levels and various CSVD phenotypes using Mendelian randomization (MR). Data on circulating omega‐3 FA levels were derived from a large genome‐wide association study (GWAS) summary dataset comprising 115,060 individuals. Data on CSVD phenotypes were obtained from several large GWAS datasets, including those on LS (6030 cases, 248,929 controls), WMH (*N* = 42,310), CMB (3556 cases, 22,306 controls), and WMPVS (9223 cases, 29,648 controls). Suitable genetic variants were extracted from GWAS summary data to serve as instrumental variables (IVs) for a two‐sample MR analysis. The primary method used was inverse variance weighted (IVW), supplemented by the weighted median (WM) and MR‐Egger method to assess causality. Multiple sensitivity analyses were used to assess the robustness of the results. Elevated circulating Omega‐3 FA levels were found to significantly reduce the risk of CMB (IVW OR = 0.82, 95% CI: 0.69–0.96). However, no significant associations were observed with WMH, WMPVS, or LS. Sensitivity analyses confirmed the stability of these results, with no indications of horizontal pleiotropy or reverse causality. This study suggests that higher circulating omega‐3 FA levels may be causally associated with a modest reduction in the risk of cerebral microbleeds. These findings highlight the potential of omega‐3 FA as a modifiable factor for preventing CMB and encourage further research in diverse populations.

## Graphical abstract

This Mendelian randomization study demonstrated that higher circulating omega‐3 fatty acid levels were causally associated with a modest reduction in risk of cerebral microbleeds (CMB). No significant associations were observed for other CSVD phenotypes, including WMH, LS, and WMPVS. These findings highlight omega‐3 fatty acids as a potential modifiable factor for CMB prevention, while emphasizing the need for validation in larger and multi‐ancestry cohorts.

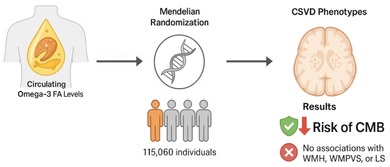

## 1 Introduction

Cerebral small vessel disease (CSVD) refers to a spectrum of clinical, imaging, and pathological syndromes caused by various factors affecting the small arteries, arterioles, capillaries, venules, and small veins in the brain (Wardlaw et al. 2019; Markus and de Leeuw 2023). The diagnosis of CSVD primarily relies on neuroimaging, with key imaging features including lacunar stroke (LS), white matter hyperintensities (WMH), white matter perivascular space (WMPVS), and cerebral microbleeds (CMB) (Duering et al. 2023; Goeldlin et al. 2022). Recent studies have shown a strong association between CSVD and heightened risks of stroke and cognitive impairment, highlighting the importance of preventing and managing CSVD to reduce the burden of stroke and cognitive decline (Wardlaw et al. 2019; Cuadrado‐Godia et al. 2018; Gorelick et al. 2017).

The pathogenesis of CSVD is multifactorial and remains incompletely understood, but emerging evidence suggests that inflammation, oxidative stress, endothelial dysfunction, and dyslipidemia play important roles in its development and progression (Adams et al. 2023; de Almeida et al. 2020; Grochowski et al. 2018; Mu et al. 2021). Given this, dietary components with anti‐inflammatory and neuroprotective effects have drawn significant attention as potential modulators of CSVD risk. Omega‐3 fatty acid (FA) has shown promising effects in cardiovascular and cerebrovascular health through mechanisms that include reducing inflammation, decreasing oxidative stress, and enhancing endothelial function (Zanetti et al. 2017; Yamagata 2020; Innes and Calder 2020; Djuricic and Calder 2021). Although observational studies generally suggest that omega‐3 FA has a protective effect against stroke and other forms of vascular diseases, the causal link between circulating omega‐3 FA levels and specific CSVD phenotypes remains unclear. Moreover, potential confounders, reverse causality, often obscure the true relationship between the two. Therefore, it is necessary to employ more rigorous research methods to clarify the relationship between circulating omega‐3 FA levels and CSVD.

Similar to natural randomized trials, Mendelian randomization (MR) is widely employed as a causal inference method to evaluate the impact of specific exposures on disease outcomes (Davies et al. 2018). By using genetic variants as instrumental variables (IVs), MR can overcome the risks of reverse causation and confounding inherent in traditional observational studies (Burgess et al. 2017). In recent years, MR has been widely utilized in epidemiological studies, demonstrating significant potential in clarifying the causal relationships between biomarkers, nutrients, and diseases. Given the increasing interest in omega‐3 FA and the potential impact on cerebrovascular health, this study seeks to clarify the causal relationship between circulating omega‐3 FA levels and various phenotypes of CSVD through Mendelian randomization.

## 2 Materials and Methods

### 2.1 Data Sources

In this MR study, single nucleotide polymorphisms (SNPs) were employed as IVs to examine the causal association between Omega‐3 FA and CSVD phenotypes. Summary data on omega‐3 FA was sourced from a large genome‐wide association study (GWAS) dataset, involving 115,060 samples and 11,590,399 SNPs (Richardson et al. 2022). This summary data is accessible through the MRC IEU OpenGWAS project database (https://gwas.mrcieu.ac.uk/). Data on CSVD phenotypes were derived from several large GWAS studies, including LS (6030 cases and 248,929 controls), WMH (*N* = 42,310), CMB (3556 cases and 22,306 controls), and WMPVS (9223 cases and 29,648 controls) (Duperron et al. 2023; Knol et al. 2020; Persyn et al. 2020; Traylor et al. 2015). Detailed information is provided in Table S1.

The operational definitions of these CSVD phenotypes were consistent with those used in the original GWAS datasets. WMH was defined as hyperintense lesions on T2‐weighted or FLAIR MRI, with total WMH volume quantified using automated image segmentation and log‐transformed for analysis. WMPVS were identified as small (< 3 mm), round or linear CSF‐isointense structures on T2‐weighted MRI, with high burden defined as subjects in the top quartile of distribution within each cohort. CMBs were defined as small (< 10 mm), round or ovoid hypointense lesions detected on susceptibility‐weighted or T2*‐GRE MRI, with cases classified according to the presence of any microbleeds. LS was defined as MRI‐confirmed small subcortical infarcts ≤ 15 mm in diameter consistent with a lacunar syndrome, after excluding cases with large artery atherosclerosis, cardioembolism, cortical infarcts, or monogenic causes. These harmonized definitions ensured consistency with prior GWAS and reflect standard criteria in CSVD research.

All the original GWAS studies had obtained the necessary ethical approvals and participant consent. This study was designed and reported in accordance with the Strengthening the Reporting of Observational Studies in Epidemiology using Mendelian Randomization (STROBE‐MR) guidelines (Au Yeung and Gill 2023).

### 2.2 Selection of IVs

**FIGURE 1:**
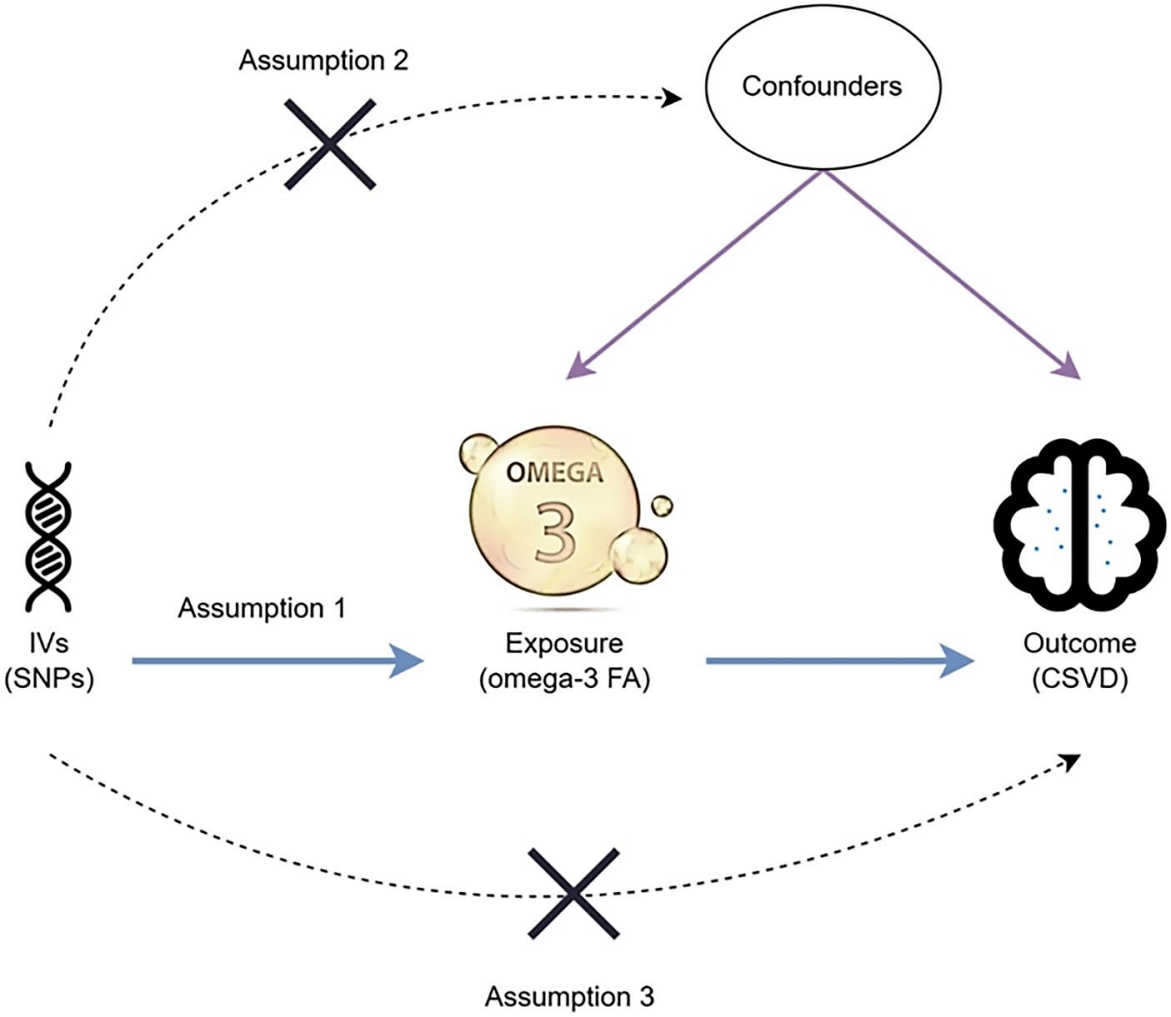
MR study design for causal relationship between omega‐3 FA and CSVD. CSVD, cerebral small vessel disease; omega‐3 FA, omega‐3 fatty acid; SNP, single nucleotide polymorphisms.

This study selects IVs based on the three core assumptions of MR (Emdin et al. 2017) (Figure 1). First, we extracted lead SNPs that were associated with circulating omega‐3 FA at genome‐wide significance (*p* < 5 × 10^−8^) from the GWAS data. To ensure independence between instruments, we performed linkage disequilibrium (LD) clumping using the 1000 Genomes European reference panel, with a clumping window of 10,000 kb and an LD threshold of *r* ^2^ < 0.001. Second, to minimize potential confounding, each candidate SNP was queried in the MRC IEU OpenGWAS database for associations with major cardiovascular risk factors, including hypertension, hyperlipidemia, obesity, diabetes, smoking, and excessive alcohol consumption. SNPs that showed genome‐wide significant associations (*p* < 5 × 10^−8^) with any of these traits were excluded to reduce the risk of violating the independence assumption. In addition, SNPs that were genome‐wide significantly associated with any of the CSVD phenotypes (WMH, CMB, LS, or WMPVS) were removed to avoid direct instrument–outcome associations. Third, we harmonized the exposure and outcome datasets so that the effect alleles were aligned in the same direction. This was done using the harmonise_data function in the TwoSampleMR package, which automatically corrects strand inconsistencies. Palindromic SNPs with intermediate allele frequencies (effect allele frequency between 0.42 and 0.58) were removed because their strand could not be reliably inferred, whereas palindromic SNPs with clearly non‐intermediate allele frequencies were retained after alignment. Finally, we calculated the *F*‐statistic for each SNP as *F* = *β* ^2^/SE^2^ and excluded SNPs with *F* < 10 to avoid weak instrument bias (Pierce et al. 2011). The remaining SNPs that fulfilled all of the above criteria were used as IVs in the primary MR analyses. For the reverse MR analyses, each CSVD phenotype was in turn treated as the exposure and circulating omega‐3 FA as the outcome. Because the number of SNPs reaching conventional genome‐wide significance for some CSVD traits was limited, we used a suggestive significance threshold of *p* < 5 × 10^−6^ to construct instruments for the reverse direction. We then used the same procedure as for the forward Mendelian randomization analysis to construct effective IVs.

### 2.3 MR Analysis

In order to analyze the assumed causal effects, inverse variance weighted (IVW) is used as the primary analytical strategy, supplemented by the weighted median (WM) method and MR‐Egger method, and applied various sensitivity analyses to account for pleiotropy (Bowden et al. 2016; Burgess and Thompson 2017). We performed several sensitivity analyses to examine pleiotropy, heterogeneity, and the influence of individual SNPs. Horizontal pleiotropy was assessed using the MR‐Egger intercept test; a non‐zero intercept indicates the presence of directional pleiotropy. We also applied the Mendelian Randomization Pleiotropy RESidual Sum and Outlier (MR‐PRESSO) method, which includes a global test for horizontal pleiotropy and identifies outlier SNPs that disproportionately drive heterogeneity (Burgess and Thompson 2017; Verbanck et al. 2018). When outliers were detected, analyses were repeated after removing these SNPs. Cochran's *Q* statistic and the corresponding *I* ^2^ index were used to quantify heterogeneity across SNP‐specific Wald ratios under the IVW framework. To further examine the robustness of the findings, we performed leave‐one‐out analyses, in which each SNP was removed in turn and the IVW estimate was recalculated, to identify whether any single SNP unduly influenced the results. Funnel plots were visually inspected to evaluate the symmetry of SNP‐specific estimates, and scatter plots and forest plots were generated to illustrate the individual and overall causal estimates for each MR analysis. Possible reverse causation was investigated using the MR Steiger directionality test, which compares the variance explained in the exposure versus the outcome to infer the most likely causal direction. In addition, we performed a reverse two‐sample MR analysis, using each CSVD phenotype as the exposure and circulating Omega‐3 FA as the outcome factor, and repeated the same MR procedure to test for the existence of reverse causality. The advantage of comparing multiple analytical methods is their ability to cross‐validate results, thereby enhancing the robustness of our conclusions.

All analyses were performed in R (version 4.4.1) using the TwoSampleMR package for data extraction, harmonization, and MR estimation, and the MRPRESSO package for pleiotropy and outlier detection. Two‐sided *p* values < 0.05 were considered statistically significant. The full R code used to implement data preparation, instrument selection, MR analyses, and figure generation is provided in File S1. Statistical power calculations were performed using the online statistical power calculator (https://sb452.shinyapps.io/power/).

## 3 Results

### 3.1 Causal Estimation in MR

**FIGURE 2:**
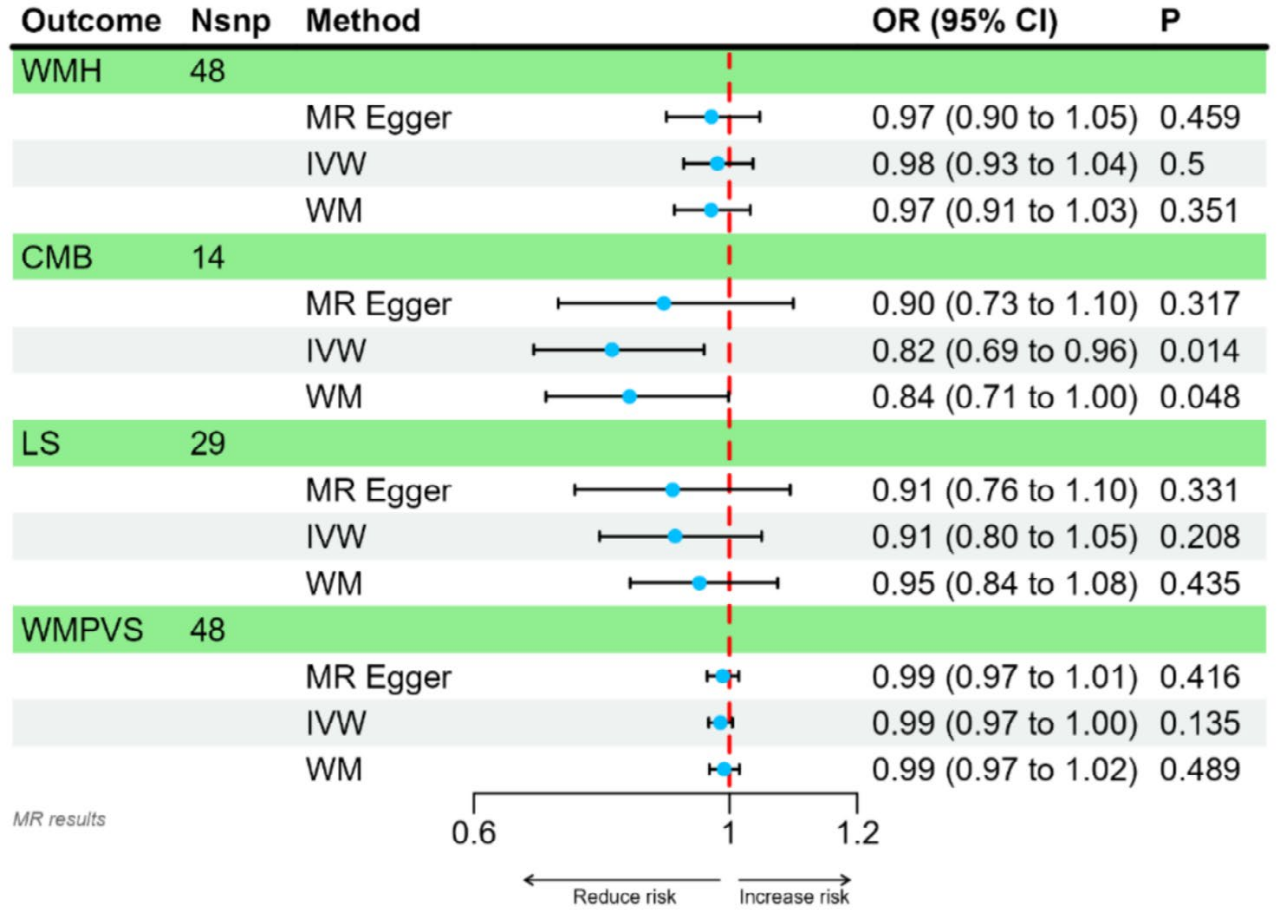
Results of MR analysis. CI, confidence interval; CMB, cerebral microbleed; IVW, inverse variance weighted; LS, lacunar stroke; Nsnp, the number of single nucleotide polymorphisms used for analysis; OR, odds ratio; WM, weighted median; WMH, white matter hyperintensity; WMPVS, white matter perivascular space.

A total of 48 SNPs were identified as IVs for WMH and WMPVS, 14 SNPs for CMB, and 29 SNPs for LS. The complete list of SNPs utilized in this study is provided in Table S2. As illustrated in Figure 2, the IVW identified a significant inverse relationship between Omega‐3 FA levels and CMB risk (OR = 0.82, 95% CI: 0.69–0.96). The WM method also demonstrated a similar causal estimate (OR = 0.84, 95% CI: 0.71–1.00). Meanwhile, MR‐Egger regression indicated a consistent direction but did not reach statistical significance (OR = 0.90, 95% CI: 0.73–1.10). Moreover, no evidence was found to suggest an association between Omega‐3 FA levels and WMH, WMPVS, or LS.

### 3.2 Results of Sensitivity Analysis

As shown in Table 1, moderate heterogeneity among SNPs was detected in the causal analysis between omega‐3 FA levels and LS (*p* = 0.016). However, the MR‐PRESSO global test did not detect any outliers (*p* = 0.096), and the MR‐Egger intercept test showed no evidence of horizontal pleiotropy (*p* = 0.945). Furthermore, the funnel plot was nearly symmetric (Figure S1), and the *I* ^2^ index showed only moderate heterogeneity (*I* ^2^ = 39.5%), suggesting that the MR results are unlikely to be significantly influenced by heterogeneity. Similarly, in all other analyses, the MR‐Egger intercept test revealed no pleiotropy (*p* > 0.05), and the MR‐PRESSO global test detected no outliers (*p* > 0.05). All IVs had *F*‐statistic > 10, indicating that our results were not influenced by weak instrument bias (Table S2). Furthermore, the leave‐one‐out analysis demonstrated that our results were not driven by any single SNP (Figure S2). The statistical power to detect was 0.78 for CMB, 0.47 for LS, 0.21 for WMH, and 0.04 for WMPVS (Table S3). These findings suggest that the null associations for WMH, LS, and WMPVS should be interpreted with caution, as they may partly reflect limited statistical power rather than a true lack of causal effect. Scatter plots provide visualization of the trend of causal effects of Omega‐3 FA on several CSVD phenotypes (Figure S3). Figure S4 provides forest plots of the effect of a single SNP on CSVD. Finally, neither the reverse MR Steiger test nor the reverse Mendelian randomization analysis provided evidence of reverse causality (Table 1 and Figures S5–S9). The results of a series of sensitivity analyses demonstrated the reliability of the results of the MR analysis.

**TABLE 1:** Results of heterogeneity test, pleiotropy test, MR‐PRESSO test, and MR Steiger test.

Outcome	Heterogeneity test			MR‐PRESSO (global test)	MR Steiger test
	*Q*	*p*	*I* ^2^	*p*	*p*
WMH	57.49	0.14	18.2%	0.201	< 0.001
CMB	11.32	0.584	0	0.497	< 0.001
LS	46.259	0.016	39.5%	0.096	< 0.001
WMPVS	41.769	0.688	0	0.716	< 0.001

Abbreviations: CMB, cerebral microbleed; LS, lacunar stroke; MR, mendelian randomization; WMH, white matter hyperintensity; WMPVS, white matter perivascular space.

## 4 Discussion

We utilized population‐scale human genetics through MR analysis to investigate the causal effects of genetically predicted circulating omega‐3 FA levels on various imaging phenotypes of CSVD. The results revealed a significant inverse causal relationship between higher circulating omega‐3 FA levels and the risk of CMB, suggesting that elevated omega‐3 FA levels may play a potential modest protective effect in reducing the risk of cerebral microvascular damage.

Our findings align with and expand upon previous studies on omega‐3 FA and vascular health. Numerous observational studies have reported potential protective effects of omega‐3 FA intake against cerebrovascular diseases. For example, a prospective cohort study involving 96,000 participants demonstrated that higher omega‐3 FA intake was significantly associated with a reduced risk of fatal stroke after adjusting for multiple confounding factors, with consistent findings across various stratified analyses (Cupino et al. 2022). Similarly, a meta‐analysis of 17 prospective cohort studies, encompassing 672,711 participants and 14,986 stroke events, reported a significant association between higher omega‐3 FA intake and reduced risks of total stroke, ischemic stroke, and hemorrhagic stroke (Chen et al. 2021). Another pooled and harmonized analysis of 29 prospective studies involving 183,291 participants with a median follow‐up of 14.3 years found that higher circulating omega‐3 FA levels were associated with a reduced risk of total stroke and ischemic stroke (O'Keefe et al. 2024). While these studies highlighted the relationship between omega‐3 FA and cerebrovascular diseases, research specifically examining the effects of omega‐3 FA on CSVD remains limited. Our study provides robust MR evidence of an inverse causal relationship between circulating omega‐3 FA levels and the risk of CMB.

The proposed mechanisms underlying the protective effects of omega‐3 FA include modulation of inflammatory responses, improvement of endothelial function, and reduction of oxidative stress. Chronic low‐grade inflammation is a key driver of CSVD, leading to endothelial dysfunction, blood–brain barrier (BBB) disruption, and microvascular injury (Wan et al. 2023; Noz et al. 2018; Fu and Yan 2018). Omega‐3FA, particularly eicosapentaenoic acid (EPA) and docosahexaenoic acid (DHA), are integral components of cell membranes and precursors to bioactive lipid mediators such as resolvins, protectins, and maresins (Maskrey et al. 2013). These specialized pro‐resolving mediators actively terminate inflammation without suppressing immune responses, distinguishing omega‐3 FA from conventional anti‐inflammatory therapies (Ishihara et al. 2019; Gutierrez et al. 2019). By mitigating inflammatory cascades and promoting the resolution of vascular inflammation, omega‐3 FA may help preserve microvascular integrity and reduce the risk of CMB.

Oxidative stress, characterized by an imbalance between reactive oxygen species (ROS) production and antioxidant defenses, damages endothelial cells and compromises the integrity of the BBB, playing a pivotal role in the development and progression of CMB (Li et al. 2023; Hannawi 2024). Omega‐3 FA exerts potent antioxidative effects through various mechanisms, including the inhibition of NADPH oxidase activity, activation of the nuclear factor erythroid 2‐related factor 2 (Nrf2) pathway, and enhancement of antioxidant enzymes such as superoxide dismutase (SOD) (Cipollina 2015; Shen et al. 2018). These actions help reduce oxidative damage to cerebral microvasculature, maintain the integrity of the BBB, and consequently decrease the occurrence of CMB. It is noteworthy that CMB are heterogeneous in etiology, arising from hypertensive arteriopathy, typically manifesting as deep CMB, and from cerebral amyloid angiopathy (CAA), which predominantly causes lobar CMB (Kuo et al. 2024; Huhndorf et al. 2023). These subtypes reflect distinct pathophysiological mechanisms. The protective association we observed may vary accordingly: omega‐3 FA could plausibly exert greater effects on hypertensive arteriopathy–related CMB by improving endothelial function, reducing inflammation, and lowering blood pressure and lipid levels, whereas their impact on CAA‐related lobar CMB is less clear and warrants further investigation. Unfortunately, the GWAS summary statistics used in our study did not distinguish CMB by etiology or anatomical distribution, preventing stratified analyses. Future large‐scale imaging genetic studies that differentiate CMB subtypes will be essential to elucidate whether the protective effects of omega‐3 FA differ across CMB etiologies.

Furthermore, several of the instrumental SNPs used in our analysis are located in or near genes with established functions in omega‐3 FA metabolism and lipid biology (Table S4). For example, variants within the FADS1/FADS2 gene cluster (e.g., rs174564, rs12226389) directly influence the desaturation of polyunsaturated fatty acids and are well‐recognized determinants of circulating omega‐3 FA levels. Other instruments map to genes regulating lipid metabolism, including ABCA1 (cholesterol efflux and HDL biogenesis), ANGPTL8/APOA1 (lipoprotein metabolism), PPP1R3B (liver glycogen/lipid metabolism), and MIR148A (post‐transcriptional regulation of LDLR and ABCA1). These loci provide biological plausibility for our findings, as lipid and lipoprotein pathways are mechanistically linked to vascular function, endothelial integrity, and inflammation—processes central to the development of CSVD and CMB. Nevertheless, not all SNPs included as instruments have known functional annotations, and many were selected based on their strong statistical association with circulating omega‐3 FA levels. Future studies integrating functional annotation, expression quantitative trait loci (eQTL) data, and pathway analyses will be important to further elucidate the biological relevance of these genetic instruments.

Importantly, statistical power analyses revealed that the study had sufficient power to detect this association for CMB, but power was more limited for LS WMH, and WMPVS. These results suggest that the null findings for WMH, LS, and WMPVS should be interpreted with caution, as they may partly reflect insufficient statistical power rather than a true absence of causal effects. Future MR analyses incorporating larger or meta‐analyzed GWAS datasets will be necessary to clarify potential associations with these phenotypes.

The strengths of this study include the use of MR design to infer causal relationships, which mitigate confounding factors and reverse causation inherent in traditional observational studies to some extent. Multiple sensitivity analyses were conducted to validate the findings and demonstrated the absence of significant horizontal pleiotropy, thereby enhancing the reliability of our results.

However, we must acknowledge certain limitations. Since our analyses were restricted to GWAS datasets derived predominantly from individuals of European ancestry, the generalizability of our findings may be limited. This restriction reflects the current availability of large‐scale summary statistics, which are disproportionately generated in European populations. Consequently, the results may not be directly applicable to non‐European populations, who differ in both allele frequencies of omega‐3 FA–related genetic variants and in the epidemiology and subtypes of CSVD. For instance, variations in dietary patterns, omega‐3 FA metabolism, and the relative contribution of hypertensive arteriopathy versus cerebral amyloid angiopathy to CSVD across populations may influence the observed associations. Future studies incorporating diverse ancestry cohorts, such as East Asian, African, and admixed populations, are warranted to validate and extend our findings, thereby enhancing their global relevance. Another limitation is that our findings have not yet been validated in an independent cohort. Although we leveraged large‐scale summary statistics from well‐established GWAS consortia, we did not have access to individual‐level data or additional cohorts to replicate our results. The lack of replication may limit the robustness and external validity of our conclusions. Future studies using large prospective cohorts will be essential to confirm our findings and to explore potential effect heterogeneity across different populations. Additionally, although the MR‐Egger intercept did not reach statistical significance, the negative intercept value (−0.019) raises the possibility of residual directional pleiotropy, indicating that, despite the ability of MR to reduce bias from known confounders, the presence of uncontrolled pleiotropy cannot be completely excluded. Specifically, some instrumental SNPs are located in or near genes involved in lipid metabolism (e.g., the FADS gene cluster, APOE), which could influence both circulating omega‐3 FA levels and cerebrovascular phenotypes. Moreover, pathways related to systemic inflammation and vascular remodeling may also represent potential sources of horizontal pleiotropy. While our sensitivity analyses mitigate this risk, future work integrating functional annotation and pathway analyses will be required to clarify the biological relevance of these variants. Finally, the relatively small sample size of the CMB GWAS (3556 cases) compared to WMH and other phenotypes may increase the risk of false positives and warrants cautious interpretation of the observed association.

## 5 Conclusion

In conclusion, this MR study provides robust evidence of an inverse causal relationship between circulating omega‐3 FA levels and CMB risk, highlighting the potential role of omega‐3 FA in preserving the integrity of cerebral small vessels. These findings support further research into omega‐3 FA as a modifiable factor for the prevention and management of CMB.

## Author Contributions

**Yaodan Zhang:** conceptualization (equal), investigation (equal), writing – original draft (equal), writing – review and editing (equal). **Lingxue Gong:** visualization (equal), writing – original draft (equal). **Xinmin Deng:** methodology (equal), writing – original draft (equal). **Jingtao Liang:** writing – review and editing (equal). **Xiaofeng Lv:** resources (equal), writing – review and editing (equal). **Rui Lai:** data curation (equal), formal analysis (equal), writing – review and editing (equal). **Meijun Liu:** project administration (equal), supervision (equal), writing – original draft (equal).

## Funding

This research was supported by the Natural Science Foundation of Sichuan Province (Grant 24NSFSC5633).

## Ethics Statement

Ethical approval and participant agreement are not necessary because each of the original GWAS has already been approved.

## Conflicts of Interest

The authors declare no conflicts of interest.

## Supporting information


**Figure S1:** Funnel plot for omega‐3 FA on CSVD.
**Figure S2:** Leave‐one‐out analysis for omega‐3 FA on CSVD.
**Figure S3:** Scatter plot of the effect of omega‐3 FA on the risk of CSVD.
**Figure S4:** Forest plot of the effect of omega‐3 FA on the risk of CSVD.
**Figure S5:** Results of reverse MR analysis.
**Figure S6:** Funnel plot for CSVD on omega‐3 FA.
**Figure S7:** Leave‐one‐out analysis for CSVD on omega‐3 FA.
**Figure S8:** Scatter plot of the effect of CSVD on the risk of omega‐3 FA.
**Figure S9:** Forest plot of the effect of CSVD on the risk of omega‐3 FA.

**File S1:** R code for Mendelian randomization analysis of circulating omega‐3 FA and CSVD phenotype.

**Table S1:** Summary of GWAS datasets used in the Mendelian randomization analysis.

**Table S2:** Details of the SNPs used for MR analysis.

**Table S3:** Statistical power of each MR analysis.

**Table S4:** Functional annotation of instrumental SNPs used for omega‐3 Fatty Acid and cerebral microbleed analysis.

## Data Availability

All GWAS summary data analyzed in this study are publicly available from the respective consortia and publications cited in the manuscript. The analysis script used in this work is provided in File S1.
