# Supplementary material for: Causal Relationship Between Circulating Omega‐3 Fatty Acid and Cerebral Small Vessel Disease: A Mendelian Randomization Study
Source: Food Sci Nutr. 2025 Dec 15;13(12):e71344. doi: 10.1002/fsn3.71344 (PMC12703811; doi:10.1002/fsn3.71344)
Supplement: Supplementary file 1 — Figure S1: Funnel plot for omega‐3 FA on CSVD. Figure S2: Leave‐one‐out analysis for omega‐3 FA on CSVD. Figure S3: Scatter plot of the effect of omega‐3 FA on the risk of CSVD. Figure S4: Forest plot of the effect of omega‐3 FA on the risk of CSVD. Figure S5: Results of reverse MR analysis. Figure S6: Funnel plot for CSVD on omega‐3 FA. Figure S7: Leave‐one‐out analysis for CSVD on omega‐3 FA. Figure S8: Scatter plot of the effect of CSVD on the risk of omega‐3 FA. Figure S9: Forest plot of the effect of CSVD on the risk of omega‐3 FA. [file FSN3-13-e71344-s006.docx]

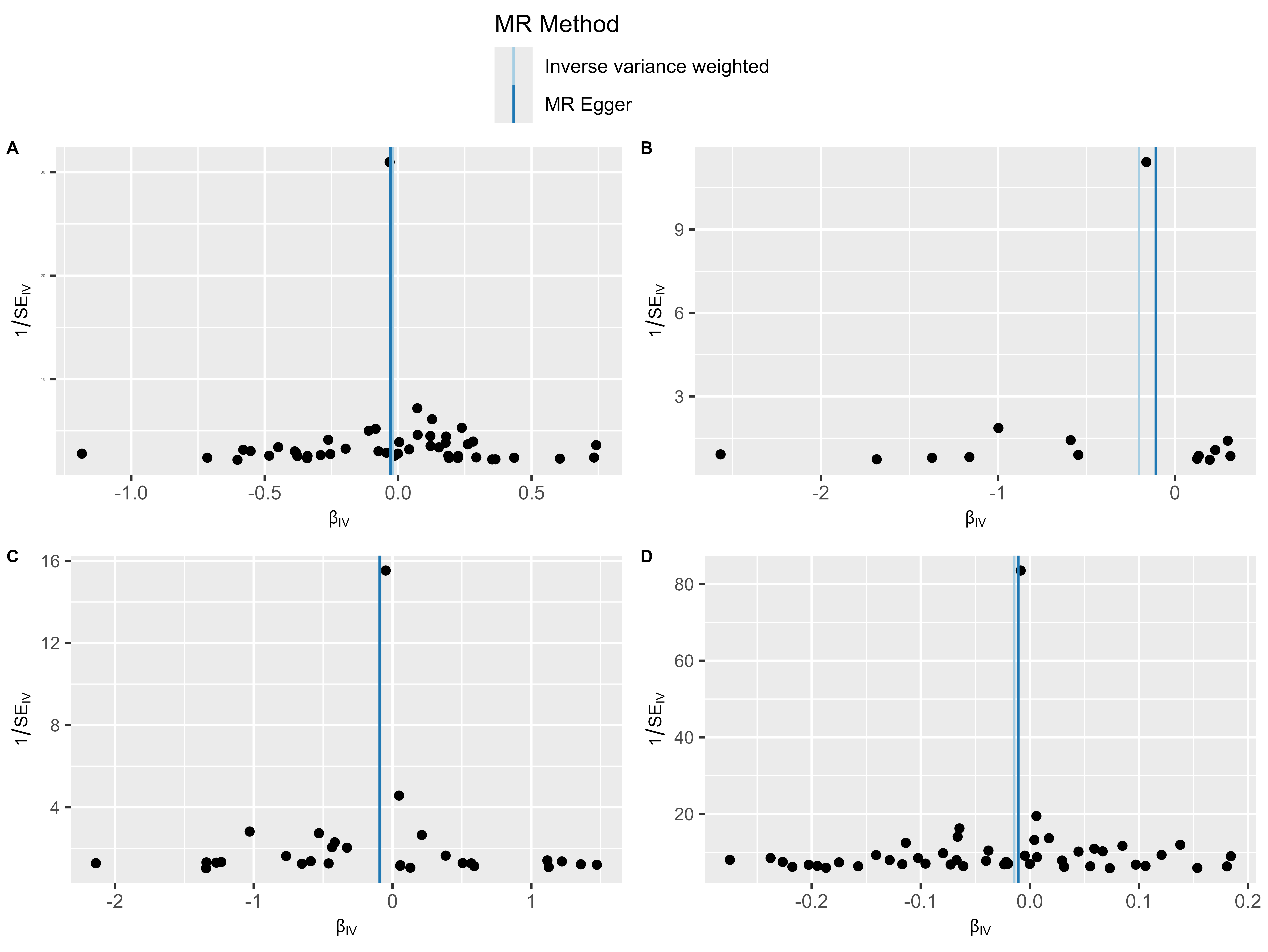
Supplementary Figure 1: Funnel plot for omega-3 FA on CSVD.


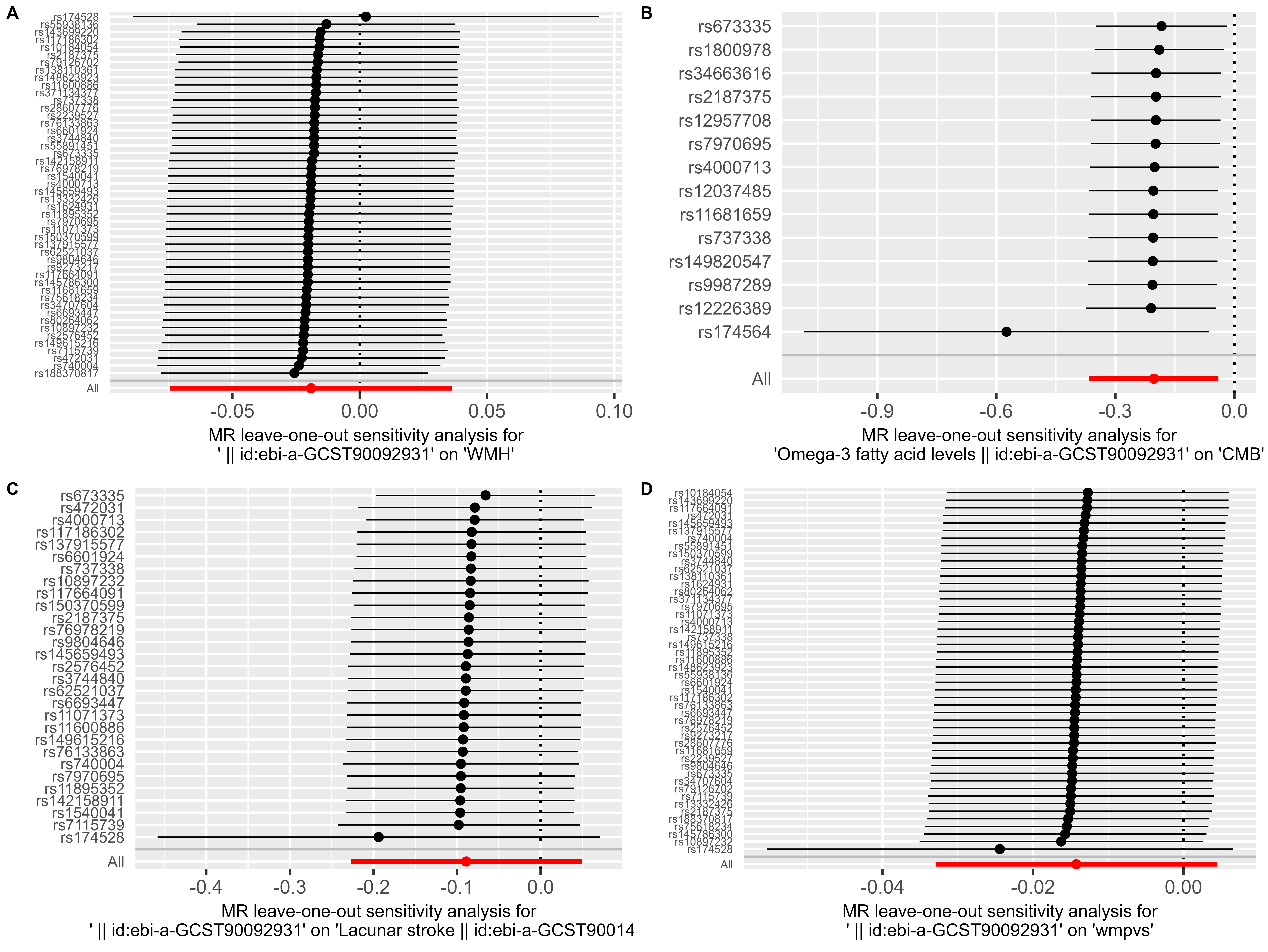


Supplementary Figure 2: Leave-one-out analysis for omega-3 FA on CSVD.

WMH, white matter hyperintensity; CMB, cerebral microbleed; LS, lacunar stroke; WMPVS, white matter perivascular space.


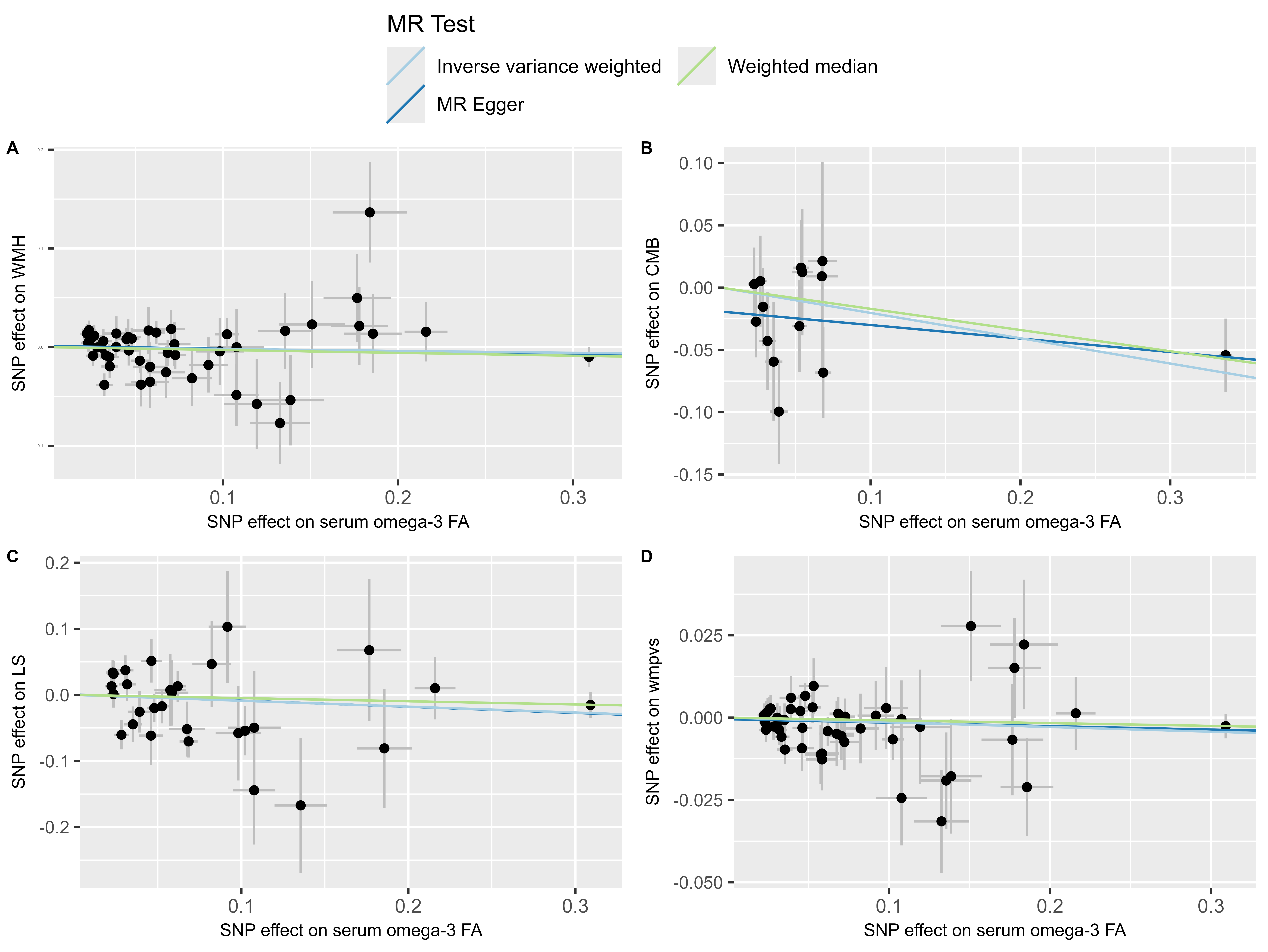


Supplementary Figure 3: Scatter plot of the effect of omega-3 FA on the risk of CSVD.


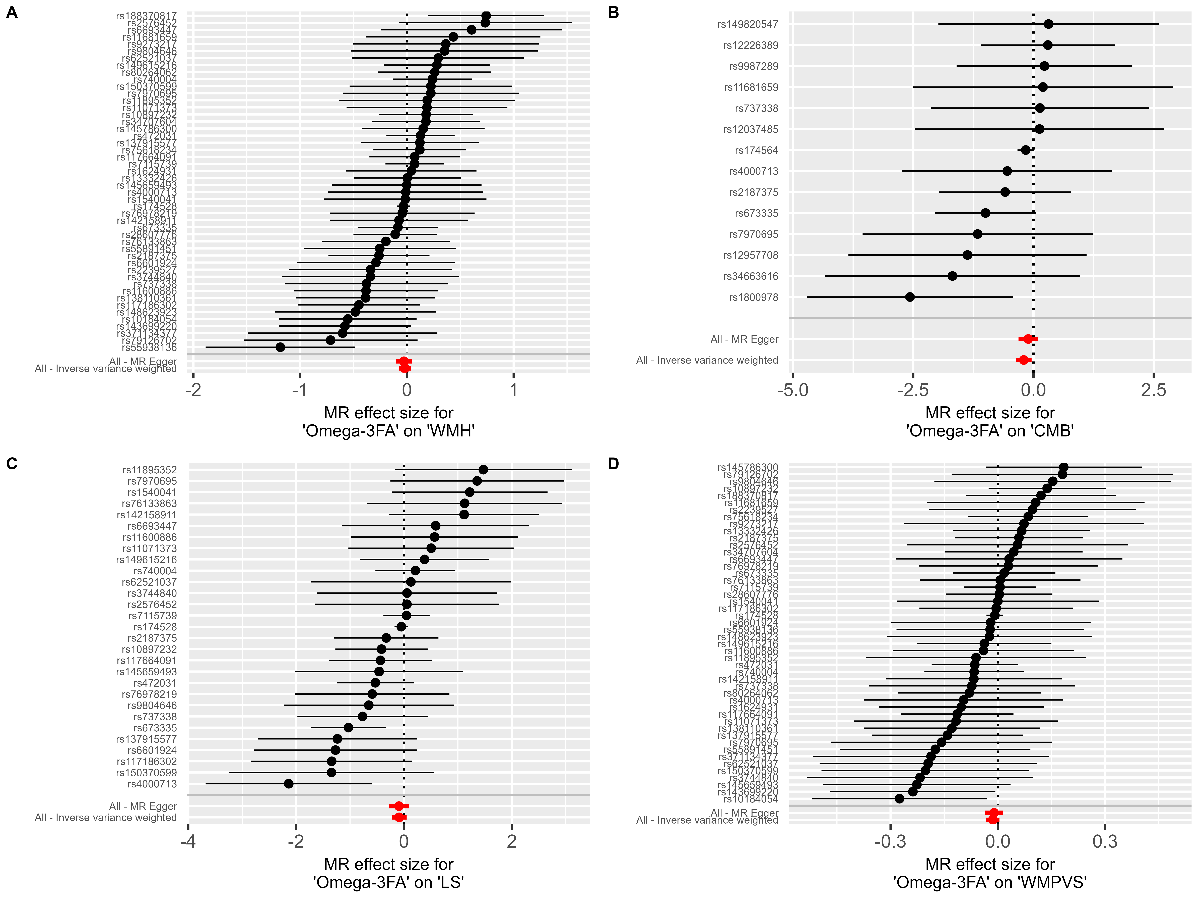


Supplementary Figure 4: Forest plot of the effect of omega-3 FA on the risk of CSVD.

WMH, white matter hyperintensity; CMB, cerebral microbleed; LS, lacunar stroke; WMPVS, white matter perivascular space.


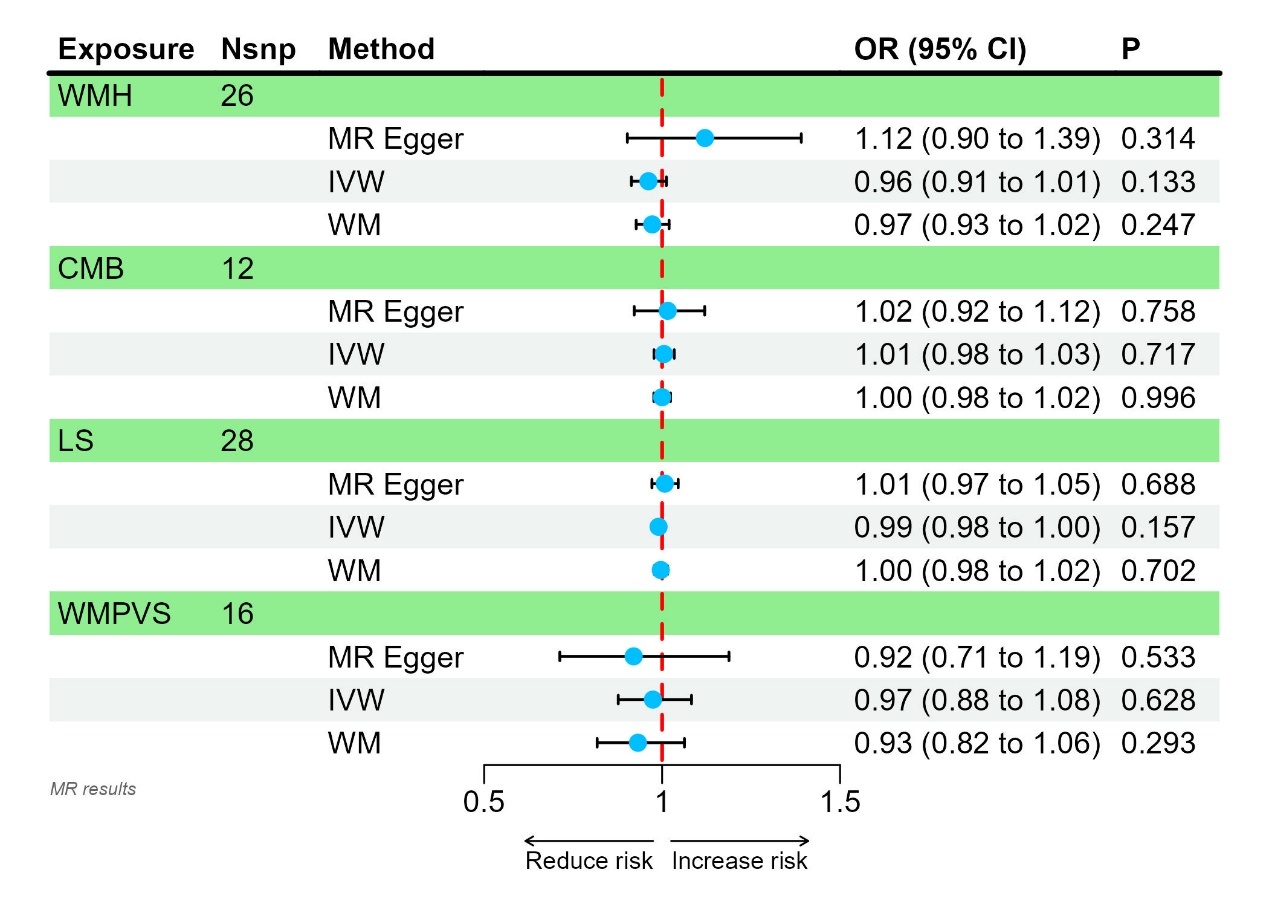


Supplementary Figure 5: Results of reverse MR analysis.

OR, odds ratio; CI, confidence interval; IVW, inverse variance weighted; WM, weighted median; Nsnp, the number of single nucleotide polymorphisms used for analysis; WMH, white matter hyperintensity; CMB, cerebral microbleed; LS, lacunar stroke; WMPVS, white matter perivascular space.


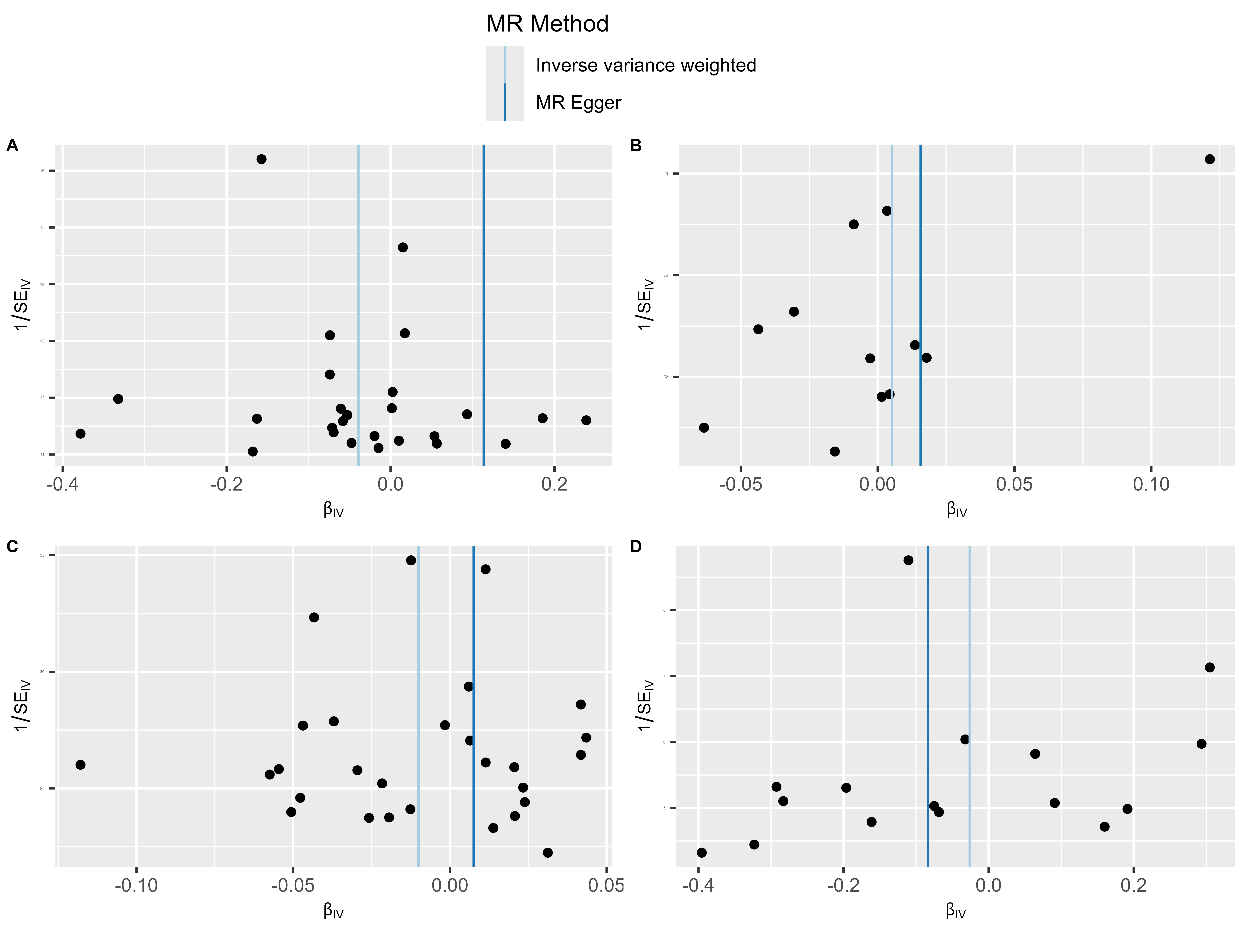


Supplementary Figure 6: Funnel plot for CSVD on omega-3 FA.


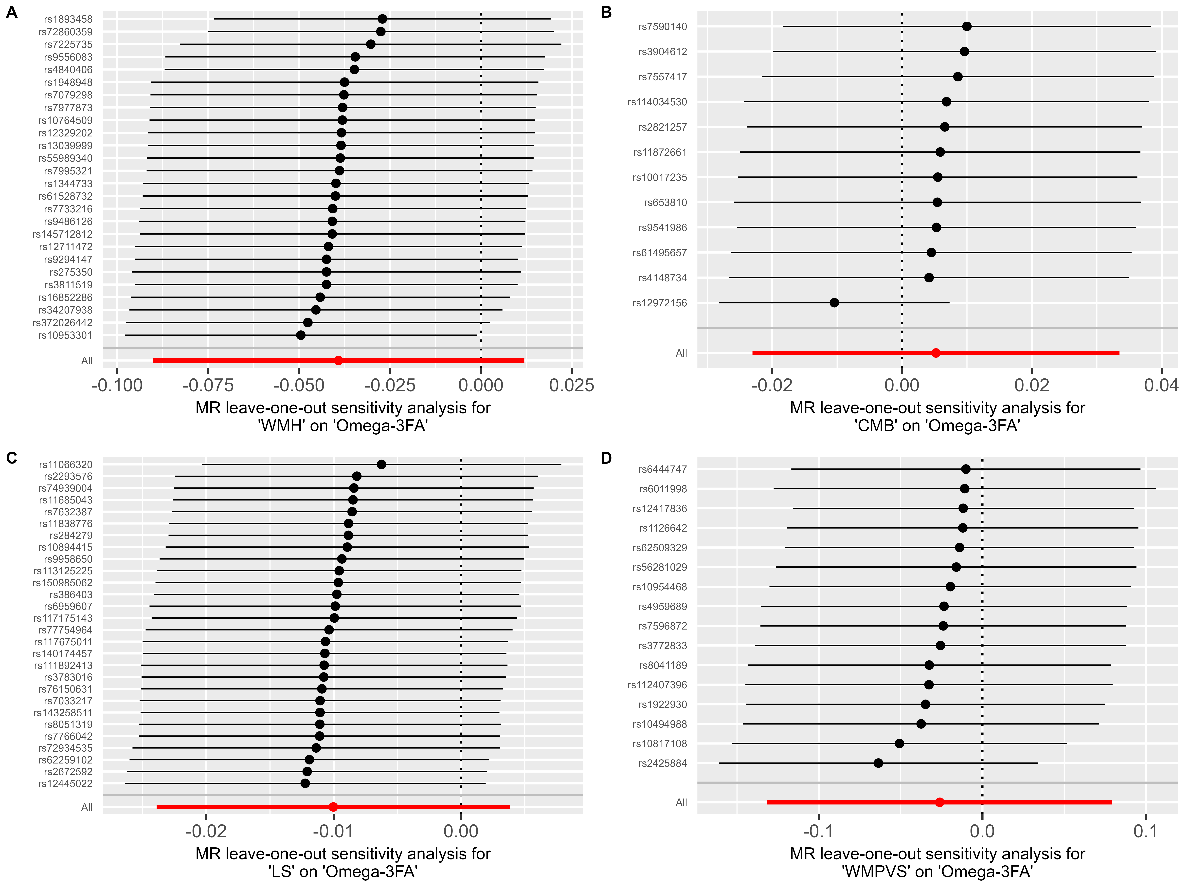


Supplementary Figure 7: Leave-one-out analysis for CSVD on omega-3 FA.

WMH, white matter hyperintensity; CMB, cerebral microbleed; LS, lacunar stroke; WMPVS, white matter perivascular space.


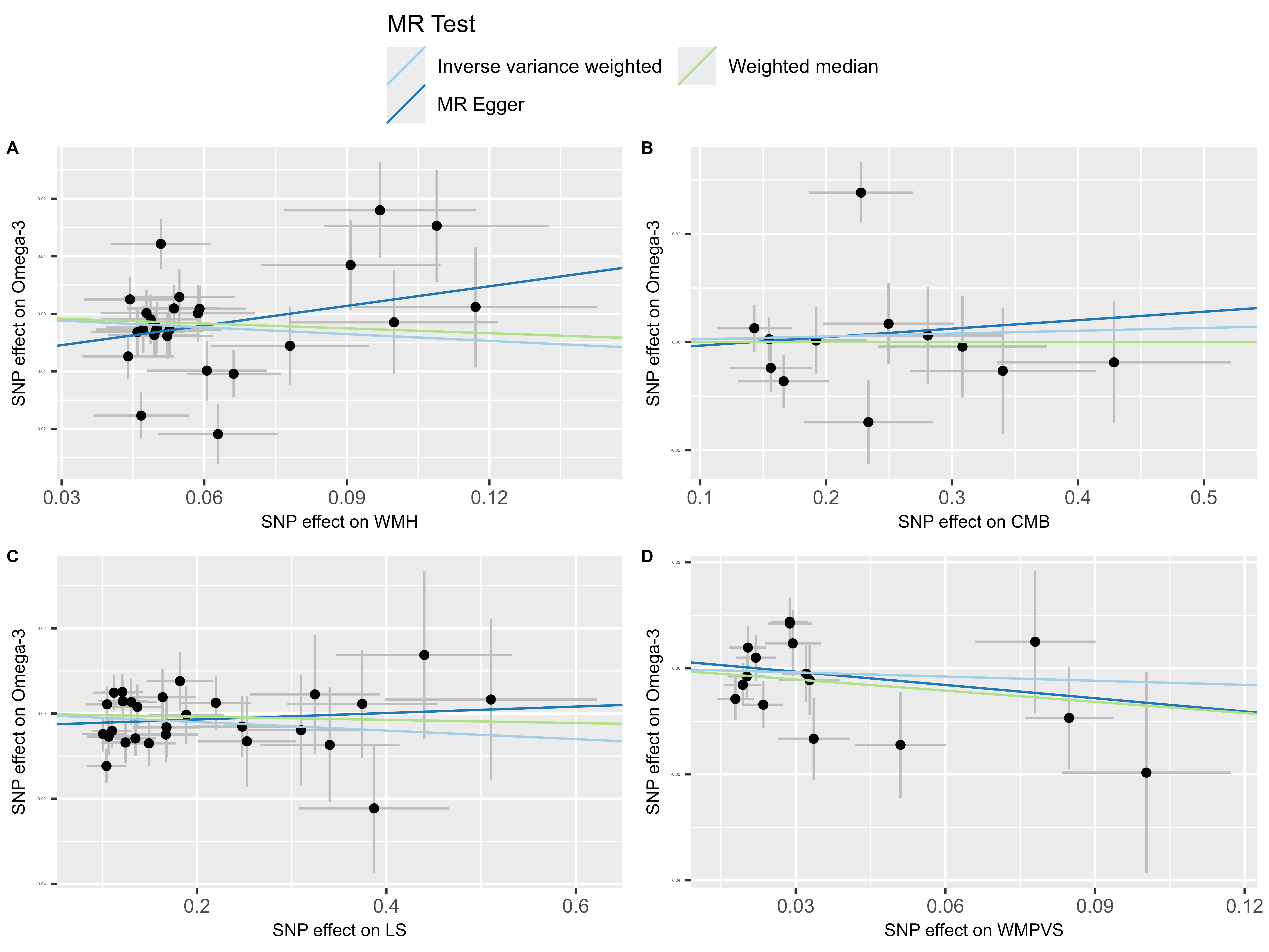


Supplementary Figure 8: Scatter plot of the effect of CSVD on the risk of omega-3 FA.


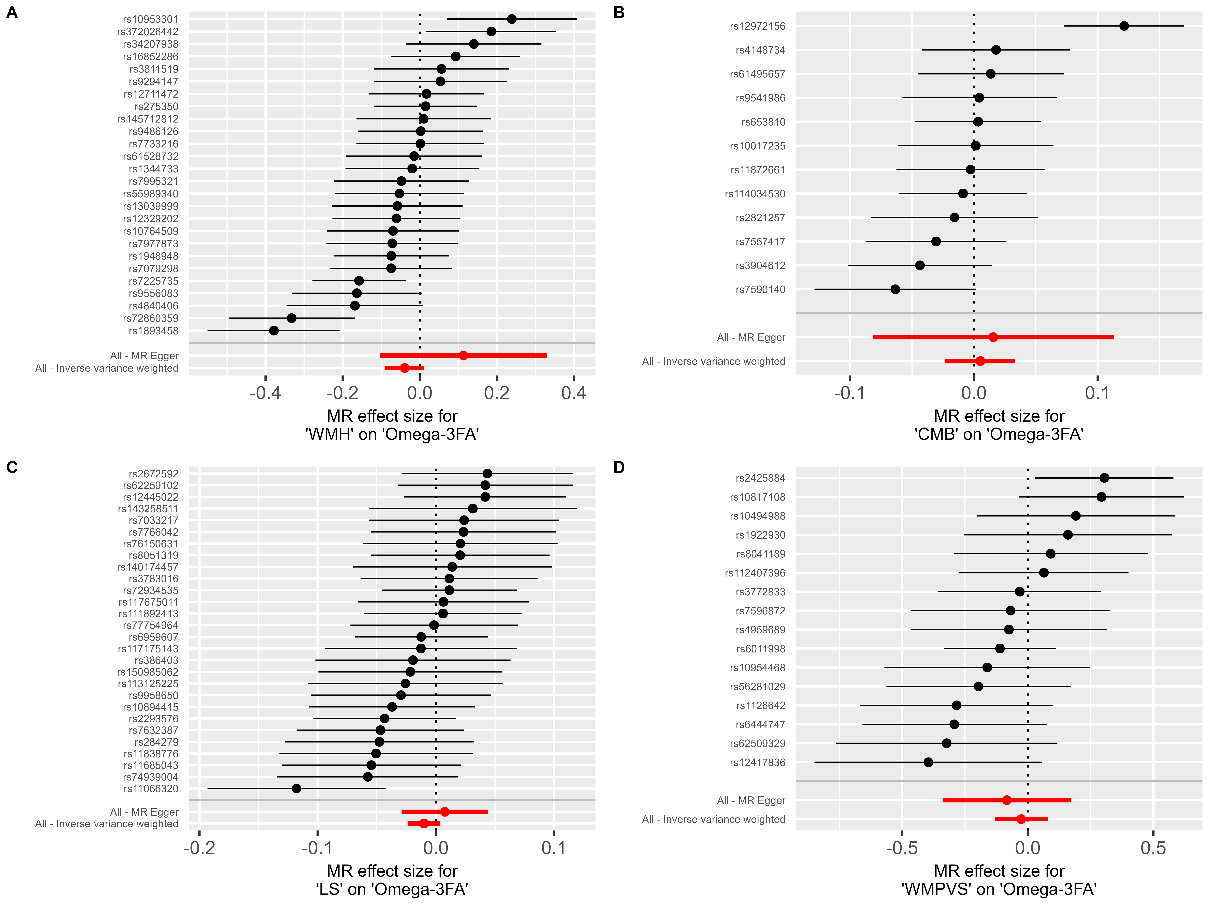


Supplementary Figure 9: Forest plot of the effect of CSVD on the risk of omega-3 FA.

WMH, white matter hyperintensity; CMB, cerebral microbleed; LS, lacunar stroke; WMPVS, white matter perivascular space.
