# Supplementary material for: Causal Relationship Between Circulating Omega‐3 Fatty Acid and Cerebral Small Vessel Disease: A Mendelian Randomization Study
Source: Food Sci Nutr. 2025 Dec 15;13(12):e71344. doi: 10.1002/fsn3.71344 (PMC12703811; doi:10.1002/fsn3.71344)
Supplement: Supplementary file 2 — File S1: R code for Mendelian randomization analysis of circulating omega‐3 FA and CSVD phenotype. [file FSN3-13-e71344-s005.docx]

Supplementary File 1. R code for Mendelian randomization analysis of circulating omega-3 FA and CSVD phenotype.

## Load required packages --------------------------------

# Install packages if necessary:

# install.packages(c("data.table", "dplyr", "ggplot2"))

# remotes::install_github("MRCIEU/TwoSampleMR")

# install.packages("devtools")

# devtools::install_github("rondolab/MR-PRESSO")

library(data.table)

library(dplyr)

library(TwoSampleMR)

library(MRPRESSO)

library(ggplot2)

## General settings --------------------------------------

# Define a function to compute F-statistics

compute_F_statistics <- function(dat) {

# beta.exposure and se.exposure are standard columns in TwoSampleMR objects

dat$F_stat <- (dat$beta.exposure ^ 2) / (dat$se.exposure ^ 2)

dat

}

############################################################

# EXPOSURE: Circulating omega-3 fatty acids

############################################################

# Exposure GWAS summary statistics file for circulating omega-3 FA

# Example format: one row per SNP with the following columns:

# SNP, beta, se, effect_allele, other_allele, eaf, pval

#

# Please replace "omega3_exposure_gwas.txt" with your actual file name

############################################################

exposure_file <- "omega3_exposure_gwas.txt"

exposure <-fread(exposure_file,header=T)

exposure_dat <- format_data(

dat= exposure,

type = "exposure",

header = TRUE,

phenotype_col = "Exposure",

snp_col = "SNP",

beta_col = "BETA",

se_col = "SE",

eaf_col = "EAF",

effect_allele_col = "EA",

other_allele_col = "OA",

pval_col = "P",

samplesize_col = "N",

id_col = "id"

)

# Select genome-wide significant SNPs (P < 5 × 10^-8)

exposure_dat <- exposure_dat %>%

filter(pval.exposure < 5e-8)

# LD clumping

# This uses the default settings in TwoSampleMR

exposure_dat_clumped <- clump_data(

exposure_dat,

clump_kb = 10000,

clump_r2 = 0.001,

pop = "EUR"

)

# 2.3 Exclude SNPs associated with major confounders

# (e.g., BMI, diabetes, lipids, smoking) by querying OpenGWAS.

# 2.4 Compute F-statistics and remove weak instruments (F < 10)

exposure_dat_clumped <- compute_F_statistics(exposure_dat_clumped)

exposure_dat_clumped <- exposure_dat_clumped %>%

filter(F_stat >= 10)

############################################################

# OUTCOMES: CSVD phenotypes

# Four outcomes:

# - Cerebral microbleeds (CMB)

# - White matter hyperintensity (WMH)

# - Lacunar stroke (LS)

# - White matter perivascular spaces (WMPVS)

#

# Each outcome is stored in a separate summary statistics file.

############################################################

# We take CMB as an example.

outcome _file <- "CMB.txt"

outcome <-fread(outcome _file,header=T)

outcome <- as.data.frame(Outcome)

Phenotype <- "CMB"

outcome$Phenotype <- Phenotype

outcome <- format_data(

dat=outcome,

type = "outcome",

header = TRUE,

phenotype_col = "Phenotype",

snp_col = "SNP",

beta_col = "BETA",

se_col = "SE",

eaf_col = "EAF",

effect_allele_col = "EA",

other_allele_col = "OA",

pval_col = "P",

ncase_col = "Ncase",

samplesize_col = "N",

chr_col = "chr",

pos_col = "pos"

)

# Harmonise exposure and outcome

dat_harmonised <- harmonise_data(

exposure_dat_clumped,

outcome_dat,

action = 2

)

# Recalculate F-stat (in case some SNPs have been removed)

dat_harmonised <- compute_F_statistics(dat_harmonised)

dat_harmonised <- dat_harmonised %>%

filter(F_stat >= 10)

# Main MR analyses: IVW, MR-Egger, Weighted median

mr_results <- mr(dat, method_list = c("mr_egger_regression", "mr_ivw","mr_weighted_median"))

# OR <-generate_odds_ratios(mr_results)

# Heterogeneity (Cochran's Q)

heterogeneity_results <- mr_heterogeneity(

dat_harmonised)

# Horizontal pleiotropy (MR-Egger intercept)

pleiotropy_results <- mr_pleiotropy_test(dat_harmonised)

# 4.7 Leave-one-out analysis

loo <- mr_leaveoneout(

dat_harmonised,

method = "mr_ivw_mre"

)

# MR-PRESSO global and outlier test

presso_input <- dat_harmonised %>%

mutate(

BetaExposure = beta.exposure,

BetaOutcome = beta.outcome,

SdExposure = se.exposure,

SdOutcome = se.outcome

)

presso_results <- tryCatch(

{

mr_presso(

BetaOutcome = "BetaOutcome",

BetaExposure = "BetaExposure",

SdOutcome = "SdOutcome",

SdExposure = "SdExposure",

OUTLIERtest = TRUE,

DISTORTIONtest = TRUE,

data = presso_input,

NbDistribution = 1000,

SignifThreshold = 0.05

)

},

error = function(e) {

message("MR-PRESSO failed for ", outcome_name, ": ", e$message)

NULL

}

)

# 4.9 Steiger directionality test

directionality <- directionality_test(dat_harmonised)

# 4.10 Generate basic plots (scatter, forest, funnel, leave-one-out)

scatter_plots <- mr_scatter_plot(mr_results, dat_harmonised)

forest_plots <- mr_forest_plot(loo)

funnel_plots <- mr_funnel_plot(

mr_singlesnp(dat_harmonised)

)

loo_plots <- mr_leaveoneout_plot(loo)
